# A Bibliographic Analysis of Primary Studies on Physical Activity and COVID-19 during 2020–2021

**DOI:** 10.3390/epidemiologia3030024

**Published:** 2022-06-22

**Authors:** Melissa Ciuldim, Rozangela Verlengia, Alex Harley Crisp

**Affiliations:** Post-Graduate Program in Human Movement Sciences, School of Health Sciences, Methodist University of Piracicaba (UNIMEP), Piracicaba 13400-911, Brazil; mel.ciuldim@hotmail.com (M.C.); rozverlengia@gmail.com (R.V.)

**Keywords:** physical inactivity, sedentary, coronavirus, pandemic, bibliometrix

## Abstract

Physical distancing and restrictions have been implemented to reduce the transmission rate of the novel coronavirus (COVID-19). In contrast, the impact of the pandemic on levels of physical activity has been the subject of studies worldwide. Since the first reported case in December 2019, the number of scientific studies on COVID-19 has grown in a way that has never been seen before. The current study aimed to perform a bibliometric analysis of primary studies on physical activity and COVID-19 during the first two years of the pandemic. The search was carried out using the SCOPUS and Web of Science databases. Our analysis identified a total of 2023 published documents from 10,199 authors, with an annual growth rate of 330% between 2020 and 2021. Open-access scientific journals were the main sources of publication, and the level of collaboration among the most influential researchers contributed to productivity. A co-occurrence analysis of the authors’ keywords indicated a high prevalence of themes related to mental health, depression, anxiety, stress, sleep, and quality of life. In conclusion, the bibliometric analysis revealed a high volume of primary studies on physical activity and COVID-19 during the first two years of the pandemic, and mental health was a much discussed topic.

## 1. Introduction

In December 2019, in the capital of Hubei province, China, a new member of the coronavirus family caused an outbreak of severe respiratory syndrome; the outbreak was named the coronavirus disease of 2019 (COVID-19). Due to the high infection rate and worldwide spread of the virus, the World Health Organization (WHO) declared COVID-19 a pandemic on 11 March 2020 [1]. Since then, non-pharmacological measures, such as physical distancing, specific restrictions, and even lockdowns, have been implemented in several countries to reduce human-to-human transmission of the virus and to prevent the healthcare system from collapsing [2].

Although these extreme precautions were necessary, the COVID-19 pandemic has had a remarkable effect on the entire organizational structure of society. For example, working from home, the closure of schools and leisure facilities, and travel restrictions have strongly affected social interactions and human mobility, further accentuating other public health problems such as mental health and obesity [3].

According to WHO, the regular practice of moderate to vigorous physical activity is essential at all stages of life in order to prevent non-communicable diseases and to improve one’s quality of life and well-being [4]. Therefore, it is crucial to understand how physical activity affects human health in order to design future public health policies.

Since the first case of COVID-19 was reported, researchers worldwide have been studying the virus and its numerous consequences for public health. The amount of available information on COVID-19 has grown exponentially, with considerably higher publication rates than any other disease in the history of science [5,6].

Scientific research activity is evaluated using a bibliometric analysis, which is defined as a set of comprehensive techniques that use quantitative tools on a large volume of data to describe the patterns and trends in a given field of knowledge [7]. Thus, we used a bibliometric analysis to better elucidate the available evidence on physical activity and COVID-19 during the first two years of the pandemic and to provide an overview of the most relevant topics from primary studies.

## 2. Materials and Methods

This was an exploratory study employing a quantitative approach to analyze peer-reviewed literature on physical activity and COVID-19. To this end, analyses were performed using studies indexed in SCOPUS and the Web of Science. SCOPUS and the Web of Science were chosen because they were considered the largest multidisciplinary databases and they provide several outputs that allow a more extensive bibliometric analysis. A topic search was performed on 31 January 2021, using the following descriptors: physical activity and COVID-19. The searches were filtered according to document type (article) and period (2020 and 2021), without language and study location restrictions.

Search outputs (BibTex [SCOPUS] and plain text [Web of Science] files) were merged into a single database after removing duplicate registers. To validate the search strategy, the search outputs were inspected by two independent researchers who selected the studies based on the following criteria: having a self-reported or objective measurement of physical activity/sedentary behavior data, regardless of the domain (occupational, domestic, transportation, and leisure time) during the COVID-19 pandemic (2020 and 2021). Reviews, comments, letters to the editor, protocols, and studies unrelated to the research topic were excluded from the database. The current study was conducted following a recently proposed guideline [7]. The following bibliometric indicators were processed in R using the bibliometrix package [8]: (a) publication-related metrics, (b) authors, (c) sources, (d) keywords, and (e) a network analysis. The number of documents by the most productive authors were reviewed individually because of conflicts with their last name.

## 3. Results

**Figure 1:**
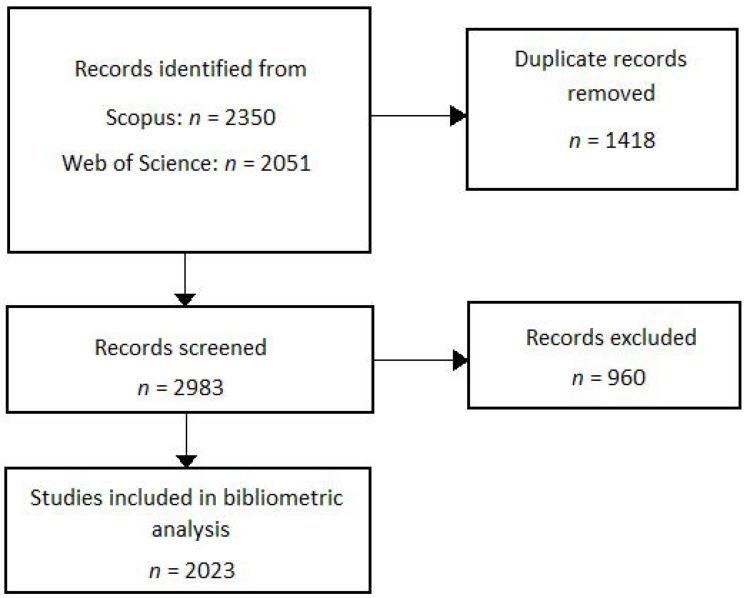
Flow chart diagram of the study search and selection process.

Figure 1 illustrates a flow chart of the studies selected for the bibliometric analysis. The search strategy identified 4401 records in electronic databases (Scopus: *n* = 2350; Web of Science: *n* = 2051), of which 1418 duplicate records were excluded. After the screening process, 960 records were not considered eligible for the bibliometric analysis. The main reasons for exclusion were: study protocols (*n* = 63); reviews, commentary articles, and letters (*n* = 416); studies that were unrelated to the research topic (*n* = 465); and studies that were published in 2022 (*n* = 16). The raw data can be found in the Supplementary Material.

An overview of the metrics on physical activity and COVID-19 is presented in Table 1. A total of 2023 documents were analyzed, with 382 studies published in 2020 and 1641 in 2021, representing an annual growth rate of 330%. The studies were published in 747 scientific journals, and had an average number of 8.7 citations per document. A total of 10,199 authors were identified, of which 10,144 were multi-authored documents. The average number of authors per document was 5.04, and the collaboration index was 5.16.

A total of 80.7% of the authors had one published document, 12.4% had two published documents, 3.7% had three published documents, and only 3.2% had four or more published documents. Deborah Carvalho Malta (Federal University of Minas Gerais, Brazil) was the most productive researcher, having co-authored 16 studies. André de Oliveira Werneck (University of São Paulo, Brazil) and Lee Smith (University of Anglia Ruskin, United Kingdom) came second, co-authoring 15 studies each (Table 2). The institutions with the most productive authors were located in Brazil (*n* = 7), Canada (*n* = 2), the United Kingdom (*n* = 2), Vietnam (*n* = 2), Austria (*n* = 1), Italy (*n* = 1), and Taiwan (*n* = 1).

Table 3 lists the 20 most prominent scientific journals that published studies on physical activity and COVID-19. In particular, the International Journal of Environmental Research and Public Health had the highest number of publications, citations, and H-index. The publication rate of the most productive journal was nearly four times higher than that of Frontiers in Psychology, which came in second. The journals located in Zone 1 of Bradford’s law clustering were considered the most prominent sources during the study period (Table 3).

**Figure 2:**
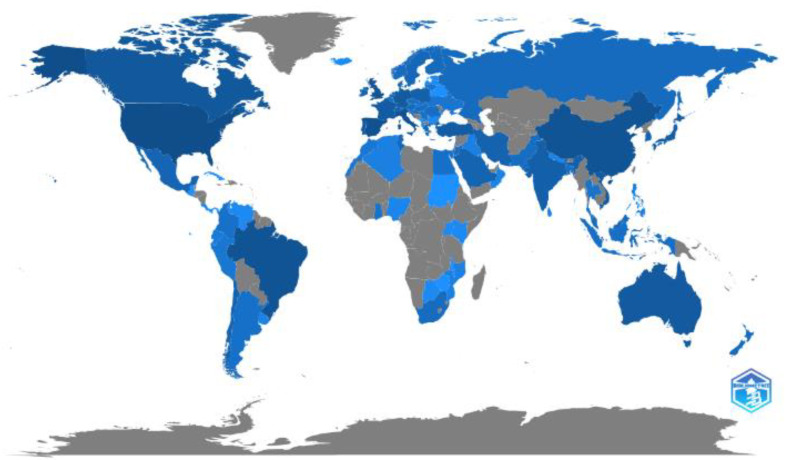
Physical activity and coronavirus disease studies (2020–2021) by country. The intensity of blue represents the publication level.

Figure 2 shows a picture of the scientific production of journal articles investigating physical activity and COVID-19 by country. Publications from 93 countries, including countries in Africa, Asia, Europe, North America, South America, and Oceania, were identified. The United States ranked first in terms of the number of published documents and citations, followed by Spain and Italy. Considering the country from which the corresponding author originates, the United States, Italy, and Spain also had the highest index of single and multiple country publications (Table 4).

The top ten most cited documents are listed in Table 5. Most of these were cross-sectional online studies conducted between February and March 2020. All studies collected self-reported measures of physical activity; two studies used the International Physical Activity Questionnaire (IPAQ) short form [9,10], one study used the Active Australia Survey [11], and one study used the Godin Leisure Questionnaire [12]. The studies were conducted in Canada [12,13], Italy [10,14], Austria [15], Australia [11], the United States [16], Switzerland [17], Vietnam [18], and multiple countries [9]. At the time of our search, the article published in the journal Nutrients titled “Effects of COVID-19 Home Confinement on Eating Behavior and Physical Activity: The results of the ECLB-COVID-19 International Online Survey” received the highest number of citations [9].

**Figure 3:**
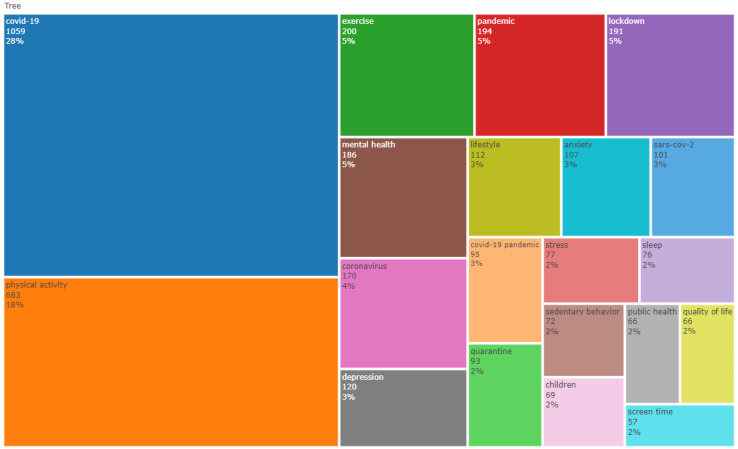
Treemap of the most prominent author’s keywords (*n* = 20).

Figure 3 shows a treemap of the authors’ keywords. The most frequent keywords were COVID-19 (*n* = 1059), physical activity (*n* = 683), exercise (*n* = 200), pandemic (*n* = 194), and lockdown (*n* = 191). The keywords relating to health outcomes included mental health (*n* = 186), depression (*n* = 120), anxiety (*n* = 107), stress (*n* = 77), sleep (*n* = 76), and quality of life (*n* = 66).

**Figure 4:**
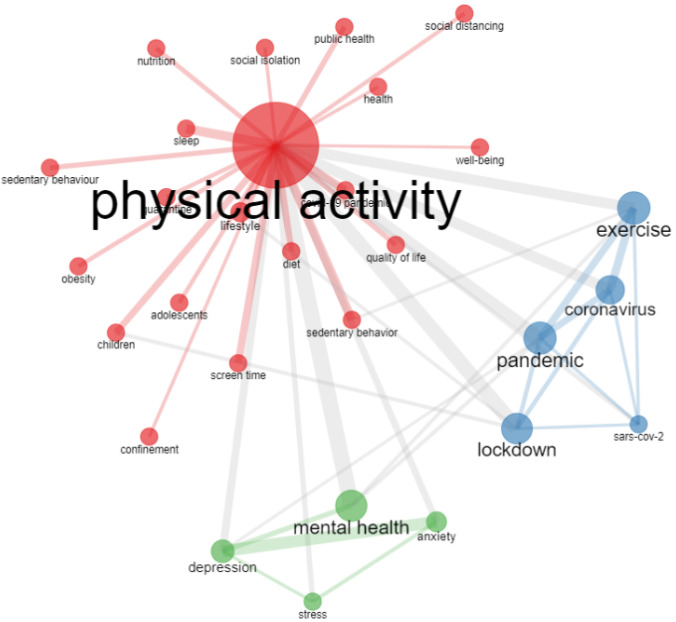
Co-occurrence network using author’s keywords (Louvain algorithm).

Figure 4 shows the co-occurrence network analysis using authors’ keywords. For better illustration, the term COVID-19 has been removed. The node size represents the frequency of occurrence, and the line size reflects the strength of the connection between keywords. The analysis, using Louvain’s algorithm and a minimum of 15 edges, resulted in the formation of three keyword clusters (red, blue, and green) based on 29 nodes.

## 4. Discussion

This study used a bibliometric analysis to summarize peer-reviewed literature on physical activity and COVID-19 during the first two years of the pandemic. Our data indicated a high volume of published primary studies, involving 10,199 authors from 93 countries. Furthermore, an exponential growth rate of annual publications became evident. This indicates that the scientific community responded rapidly in order to understand the impacts of COVID-19 on physical activity and other health-related factors during 2020 and 2021.

The COVID-19 pandemic has mobilized scientists around the world and has resulted in an increase in the number and rate of peer-reviewed articles published, whilst subsequently decreasing the number of non-COVID-19-related articles published in leading health science journals [19]. This phenomenon of an exponential increase in COVID-19 publications may be linked to several reasons:

(a). urgency for thematic research;
(b). targeted research grants;
(c). more significant publicity;
(d). specific journal issues; and
(e). decreased peer review time.

Thus, a relevant element in the publication of research investigating physical activity and COVID-19 was the expressive participation of online open-access journals. Our study found that the most influential sources of physical activity and COVID-19 studies allowed free access to academic articles (Table 3), which is important because open-access journals allow greater dissemination of scientific knowledge with equality. Some open-access journals promise a swift publication process, which may have been one of the main factors contributing to the high publication rate, as seen in the Journal of Environmental Research and Public Health.

It should be noted that collaboration networks are one of the main driving forces among the most productive researchers. For example, Brazilian researchers (Malta, Werneck, Szwarcwald, da Silva, Barros, Azevedo, de Souza Júnior) co-authored 13 studies based on data from an online national health survey (ConVid—Behavior Research) in adults and adolescents. Researchers from the United Kingdom (Smith and Tully) and Austria (Grabovac) co-authored eight studies that were conducted in the United Kingdom, Austria, Brazil, and Spain. Researchers from Vietnam (Do and Nguyen) and Taiwan (Van Duong) co-authored 10 studies with data from a population recruited from hospitals and health centers in Vietnam.

Similarly, the study that received the highest number of citations (ECLB-COVID-19 project) involved collaboration among researchers from Europe, North Africa, West Asia, and the Americas, and was designed to evaluate behavioral and lifestyle changes during the COVID-19 outbreak (1–11 April 2020) [9]. The study released an online questionnaire in 14 languages (English, German, French, Arabic, Spanish, Portuguese, Slovenian, Dutch, Persian, Italian, Greek, Russian, Indian, and Malayalam) [9].

The second most cited study [14] investigated the impact of the COVID-19 pandemic on eating habits and lifestyle changes (smoking habits, hours of sleep, frequency/type of physical activity before and during the pandemic) among the Italian population via an online survey (between 5 and 24 April 2020) during a lockdown period [14]. Although our study identified a considerable number of countries publishing research (Figure 2), Spain and Italy stood out in terms of research on physical activity during the pandemic, outperforming general high-ranking countries, including China and the United Kingdom, according to the Scimago country rank.

The treemap chart (Figure 3) of the authors’ keywords indicated that mental health and depression were the most investigated topics and had an impressive six-fold growth rate between 2020 and 2021. Furthermore, the network analysis indicated the presence of two specific clusters (mental health [green] and exercise [blue]) and a larger generic cluster involving themes such as sedentary behavior, screen time, obesity, sleep, well-being, quality of life, and lifestyle (Figure 4). These data highlight that research investigating physical activity during the pandemic was broad and dealt with multiple health issues.

This study has some limitations that need to be addressed. First, our analysis was limited to journals indexed in the SCOPUS and Web of Science databases. Therefore, we were unable to access all the available evidence. Second, we considered only primary studies (original articles), and the publication patterns of theoretical documents (e.g., comments, letters to the editor, narrative reviews, and book chapters) might be different. The main focus of the present study was to map the large amount of published research on physical activity during the first two years of the COVID-19 pandemic. On the other hand, more studies (systematic and scoping reviews) are needed to explore how COVID-19 has affected physical activity and the topics highlighted in our bibliometric analysis in greater depth.

## 5. Conclusions

In conclusion, a high rate of publication of primary studies was observed during the first two years of the pandemic, and factors such as collaborative networks between researchers and open-access journals were important pillars to leverage scientific evidence in a short period. Our findings revealed that mental health was a much discussed topic and a pressing health problem impacted by the lack of adequate physical activity during the COVID-19 pandemic.

## Figures and Tables

**Table 1:** Summary of bibliometric indicators on physical activity and coronavirus disease (2020–2021).

Description	Results
Total Documents	2023
Documents Published in 2020	382
Documents Published in 2021	1641
Sources	747
Average Citations per Document	8.7
References	58,950
Author’s Keywords	3215
Authors	10,199
Authors of single-authored documents	59
Authors of multi-authored documents	10,144
Authors per Document	5.04
Co-Authors per Document	6.8
Collaboration Index	5.16

**Table 2:** Most productive authors on physical activity and coronavirus disease (2020–2021).

Rank	Author Name	Articles (*n*)	Affiliation
1	Malta, DC	16	Federal University of Minas Gerais
2	Werneck, AO	15	University of Sao Paulo
2	Smith, L	15	Anglia Ruskin University
3	Szwarcwald, CL	13	Oswaldo Cruz Foundation
3	da Silva, DRP	13	Federal University of Sergipe
3	Azevedo, LO	13	Oswaldo Cruz Foundation
3	Barros, MBA	13	State University of Campinas
4	de Souza Júnior, PRB	13	State University of Campinas
4	Tully, MA	11	Ulster University
5	Bragazzi, NL	11	York University/University of Genoa
5	Do, BN	10	Vietnam Military Medical University
5	Grabovac, G	10	Medical University of Vienna
5	Vanderloo, LM	10	University of Western Ontario
5	Nguyen, TTP	10	Hue University of Medicine and Pharmacy
5	Van Duong, T	10	Taipei Medical University

**Table 3:** Most productive journals on physical activity and coronavirus disease (2020–2021).

Sources	Articles	Citations	Bradford’s Law Zone
*Int. J. Environ. Res. Public Health*	284	26	1
*Front. Psychol.*	77	12	1
*Nutrients*	68	18	1
*Sustainability*	40	10	1
*refPLoS ONE*	36	10	1
*J. Phys. Educ. Sport*	35	5	1
*BMC Public Health*	33	8	1
*Front Public Health*	30	4	1
*BMJ Open*	26	7	1
*J. Clin. Med.*	22	6	1
*Front Sports and Act Living*	18	3	2
*Healthcare*	17	2	2
*Front. Psychiatry*	16	7	2
*J. Med. Internet Res.*	15	9	2
*J. Human Sport Exerc.*	13	2	2
*J. Phys. Act. Health*	12	3	2
*Children* (Basel)	11	6	2
*Diabetes Metab. Syndr.*	11	7	2
*Prev. Med. Rep.*	11	4	2
*Progress In Nutrition*	11	2	2

**Table 4:** Top 10 countries with the highest level of scientific production in physical activity and coronavirus disease (2020–2021).

Country	Total Articles	Total Citations	Corresponding Author’s Country		
			Articles	SCP	MCP
USA	832	2338	243	208	35
Spain	604	1625	149	118	31
Italy	519	2072	150	122	28
UK	473	1330	134	104	30
China	458	1142	109	65	44
Brazil	426	601	108	83	25
Canada	275	1256	60	45	15
Germany	241	1160	79	61	18
Australia	239	822	54	38	16
Turkey	188	161	78	76	2

**Legend:** USA, United States of America; UK, United Kingdom; SCP, single country publications; MCP, multiple country publications.

**Table 5:** Top 10 documents with the highest number of citations on physical activity and corona-virus disease (2020–2021).

Author (Year)	Article Title	Source	Citations (January 2022)
Ammar et al. (2020) [9]	Effects of COVID-19 Home Confinement on Eating Behaviour and Physical Activity: Results of the ECLB-COVID19 International Online Survey	*Nutrients*	539
Di Renzo et al. (2020) [14]	Eating habits and lifestyle changes during COVID-19 lockdown: An Italian survey	*J. Transl. Med.*	381
Stanton et al. (2020) [11]	Depression, Anxiety and Stress during COVID-19: Associations with Changes in Physical Activity, Sleep, Tobacco and Alcohol Use in Australian Adults	*Int. J. Environ. Res. Public Health*	349
Shechter et al. (2020) [16]	Psychological distress, coping behaviors, and preferences for support among New York healthcare workers during the COVID-19 pandemic	*Gen. Hosp. Psychiatry*	285
Pieh et al. (2020) [15]	The effect of age, gender, income, work, and physical activity on mental health during coronavirus disease (COVID-19) lockdown in Austria	*J. Psychosom. Res.*	249
Moore et al. (2020) [13]	Impact of the COVID-19 virus outbreak on movement and play behaviours of Canadian children and youth: a national survey	*Int. J. Behav. Nutr. Phys. Act*	246
Maugeri et al. (2020) [10]	The impact of physical activity on psychological health during COVID-19 pandemic in Italy	*Heliyon*	232
Shanahan et al. (2020) [17]	Emotional distress in young adults during the COVID-19 pandemic: evidence of risk and resilience from a longitudinal cohort study	*Psychol. Med.*	211
Lesser et al. (2020) [12]	The Impact of COVID-19 on Physical Activity Behavior and Well-Being of Canadians	*Int. J. Environ. Res. Public Health*	196
Nguyen et al. (2020) [18]	People with Suspected COVID-19 Symptoms Were More Likely Depressed and Had Lower Health-Related Quality of Life: The Potential Benefit of Health Literacy	*J. Clin. Med.*	179

## Data Availability

Not applicable.

## Supplementary Materials

The following supporting information can be downloaded at: https://www.mdpi.com/article/10.3390/epidemiologia3030024/s1.
